# Multi-Modal Cannabis Use Among U.S. Young Adults: Findings from the 2022 and 2023 BRFSS in 23 States

**DOI:** 10.3390/ijerph22040495

**Published:** 2025-03-26

**Authors:** Nayoung Kim, Sarah Flora, Casey Elizabeth Macander

**Affiliations:** 1 Department of Health Science, University of Alabama, Tuscaloosa, AL 35487, USA; sflora@crimson.ua.edu; 2 Institute for Rural Health Research, University of Alabama, Tuscaloosa, AL 35401, USA; cemacander@crimson.ua.edu

**Keywords:** multi-modal cannabis use, sociodemographic factors, physical health, substance use, cannabis legalization

## Abstract

Cannabis use among young adults in the U.S. has nearly doubled in recent years, driven by diverse methods of consumption and evolving cannabis legalization. Multi-modal cannabis use among young adults is an emerging public health issue that remains underexplored. This study examines the prevalence, patterns, and predictors of multi-modal cannabis use, defined as the use of two or more administration methods of cannabis use (e.g., smoking, vaping, edibles, dabbing, other forms) in the past month, among U.S. young adults aged 18–34 years. Data from the 2022–2023 Behavioral Risk Factor Surveillance System (BRFSS) across 23 states (n = 7635; weighted n = 7,482,134) show that 57% of young adults reporting current cannabis use engaged in multi-modal use, with dual- and triple-mode use being the most common. Factors associated with higher odds of multi-modal use include sexual minority status, poor physical health, frequent cannabis use, and co-use of electronic cigarettes and alcohol. Recreational cannabis legalization (RCL) is significantly linked to higher odds of multi-modal use. These findings underscore the interplay between individual risk factors and cannabis policy environments in shaping multi-modal cannabis use behaviors. To mitigate potential harms, targeted prevention strategies should prioritize young adults at risk for cannabis use, addressing both personal and policy-related factors influencing multi-modal cannabis use.

## 1. Introduction

Cannabis use among young adults has emerged as a significant public health concern in the U.S. The 2024 Monitoring the Future Study found that the prevalence of past 30-day cannabis use among young adults has doubled, increasing from 14.9% in 2005 to 28.7% in 2023 [1]. While recent trends indicate stabilization, young adults continue to have the highest rates of cannabis use, identifying them as one of the most vulnerable adult subpopulations of cannabis use [1,2]. This persistent high rate of cannabis use among young adults raises substantial concerns due to its association with numerous adverse health and social outcomes such as cardiovascular and respiratory diseases [3,4], mental health disorders [5], cognitive impairments [6], and diminished performance [7].

The legalization and commercialization of cannabis in the U.S. have significantly increased the availability of various cannabis products (e.g., dried flowers, concentrates, and topicals) and expanded the methods of administration (e.g., smoking, vaping, eating/drinking, and dabbing), leading to increase the likelihood of young adults using multiple methods to consume cannabis [8,9,10]. Emerging evidence suggests that multi-modal cannabis use, defined as the use of two or more methods of administration to consume cannabis within a specified time period (e.g., in the past month) [9,10], has become more prevalent among young adults [9,10,11,12,13]. Studies with young adult samples indicate that 57–88% of those who use cannabis report engaging in multi-modal use, regularly consuming cannabis through at least two methods [10,12]. Another study found that young adults aged 18–34 years had the highest rate of multi-modal cannabis use among all adult age subgroups [13]. Furthermore, a daily diary study on day-level cannabis use found that young adults who have more cannabis use days on average are more likely to report multi-modal use on those days [11]. This multi-modal cannabis use is associated with higher frequency of use [11], greater dependence, and increased use of other substances [12,14], conferring elevated risks of cannabis-related harms compared to single-modal use [12,15,16]. However, research has not kept pace with the rapid diversification of cannabis consumption methods, leaving gaps in understanding the patterns and factors of multi-modal use among young adults in the U.S. While prior studies have documented high rates of multi-modal cannabis use and its risk factors, they are constrained by small sample sizes [10,11,12], restricted geographic coverage (e.g., data from only six areas) [10], outdated datasets (e.g., data from 2016) [9], and a narrow focus on specific demographic factors (e.g., biological sex, race/ethnicity) [9,10,13]. Thus, a deeper understanding of the prevalence and factors that increase or mitigate multi-modal use is essential for developing effective prevention strategies tailored to young adults at high risk of cannabis use.

Cannabis use patterns, including multi-modal use, vary significantly across sociodemographic subpopulations in the U.S. [2,9,10,13,17,18,19]. Higher prevalence rates of cannabis use have been observed among men, non-Hispanic White individuals, unmarried individuals, those with lower educational attainment, and sexually minoritized groups (e.g., gay, lesbian, or bisexual) [2,17,18,19]. Studies on multi-modal cannabis use have found similar trends, with higher rates of multi-modal cannabis use among men and sexually minoritized groups, while African American adults who use cannabis report a lower likelihood of multi-modal use [10,13,18]. Despite these variations in cannabis use patterns, limited research has explored how sociodemographic factors influence multi-modal cannabis use, particularly among young adults. As awareness of these disparities grows, it is important to further investigate the role of sociodemographic factors in shaping multi-modal use among young adults.

Additionally, existing research has largely overlooked the relations between modes of cannabis use, and health status and other substance use behaviors, such as the co-use of cannabis with tobacco and alcohol. While prior studies have documented that co-use of these substances is common and associated with greater risks of cannabis use (e.g., increased frequency and quantity of use) and cannabis-related disorders [20,21,22], limited attention has been given to how these co-use behaviors influence multi-modal cannabis use. Furthermore, mental or physical health status may drive cannabis use and increase the risk of cannabis dependence. For example, individuals experiencing stressful events or emotional difficulties or physical chronic pain are more likely to engage in frequent or heavy cannabis use [23,24]. However, the relations between cannabis use and physical health remains inconsistent and underexplored among young adults. While some studies report no strong association between poor physical health and cannabis use [25], others suggest that individuals in poorer physical health may be more likely to use cannabis [23]. These evidence from studies on cannabis use suggest that factors related to mental and physical health status, and co-occurring substance use behaviors likely shape patterns of multi-modal cannabis use, but these relations have yet to be thoroughly examined among young adults.

Cannabis legalization adds an additional layer of complexity to multi-modal cannabis use among young adults, with medical cannabis legalization (MCL) and recreational cannabis legalization (RCL) having distinct effects on cannabis use patterns [26,27,28,29]. For example, RCL is associated with increase in past-month cannabis use, more frequent and intense use, and changes in administration method of cannabis use [27,28,29]. However, a meta-analysis study showed that MCL has a null effect on cannabis use among young adults [26]. Young adults who use cannabis frequently are more likely to adopt multiple methods of consumption, such as combining smoking with vaping or edibles [10,11]. This suggests that cannabis legalization, especially RCL, may promote multi-modal cannabis use. Despite the documented heterogeneous effects of RCL and MCL on general cannabis use patterns, their specific impact on multi-modal cannabis use remains understudied, warranting the research to understand how RCL and MCL independently influence multi-modal cannabis use among young adults.

The current study sought to advance the literature on cannabis use prevention and control by examining the role of diverse individual and cannabis policy contextual factors in predicting multi-modal cannabis use among the U.S. young adults. This study conducts secondary analysis with data from nationally representative U.S. samples to estimate the prevalence and patterns of multi-modal cannabis use among young adults and identify associations with a range of variables, including sociodemographic characteristics, mental and physical health status, substance use behaviors (such as cannabis use frequency, cigarette smoking, smokeless tobacco, electronic cigarette use, and alcohol use) and cannabis use-related policies, including MCL and RCL. The findings from this study are intended to provide evidence that inform the development of targeted prevention strategies related to cannabis use for young adults at the high risks of cannabis-related harms.

## 2. Material and Methods

### 2.1. Data Source and Sample

This study utilized the combined cross-sectional data from 2 waves of the 2022 and 2023 Behavioral Risk Factor Surveillance System (BRFSS) in 23 states, focusing on individuals who reported cannabis use and mode type of use. The BRFSS collects data annually on health-related risk behaviors, chronic conditions, and preventive service utilization through telephone surveys of noninstitutionalized civilians aged 18 and older. The survey uses random digit dialing for both landlines and cell phones across all 50 states, the District of Columbia, and four U.S. territories, ensuring a representative sample for each state and geographic area. Participation in the survey is voluntary and anonymous. The BRFSS collects data on cannabis use from core modules and optional models adopted by some states. Data relevant on cannabis use were available for 23 states in the 2022 and 2023 BRFSS datasets: Connecticut, Delaware, Hawaii, Illinois, Indiana, Kansas, Maine, Maryland, Massachusetts, Michigan, Mississippi, Montana, Nebraska, Nevada, New Mexico, North Dakota, Ohio, Oklahoma, Oregon, Vermont, Virginia, Wisconsin, Wyoming. This study utilized state data from 16 states participating in the core module and 6 states (Kansas, Maryland, Massachusetts, Michigan, Ohio, and Oklahoma) using optional cannabis use modules in the 2022 BRFSS, and 14 states in the core module and 3 states (Maryland, Ohio, and Oklahoma) in optional cannabis use modules in the 2023 BRFSS. Data from the U.S. territories, including Guam and the Virgin Islands, were excluded from the analysis due to their relatively small sample sizes in comparison to other U.S. states, which could potentially affect the representativeness and generalizability of the findings. The analytic sample comprised a total of 7635 (weighted n = 7,482,134) young adults aged 18–34 years who reported cannabis use in the past 30 days and at least one mode of use (e.g., smoking, vaping, eating or drinking, dabbing, and other). The analytic sample included individuals aged 18–34, in accordance with the U.S. Census criterion for young adults [30], as this group has demonstrated high rates of cannabis use and multi-modal use in previous studies [1,2,13]. Additional methodological details about the BRFSS are available elsewhere (https://www.cdc.gov/brfss/data_documentation/index.htm (assessed on 2 March 2025)). The BRFSS dataset is de-identified and publicly available, and this secondary data analysis was deemed exempt by the University of Alabama’s Institutional Review Board.

### 2.2. Measures

Cannabis use in the past 30 days was assessed with the question, “During the past 30 days, on how many days did you use marijuana or hashish?” Participants who reported using cannabis on 1–30 days were classified as those who currently use cannabis, based on prior studies [31]. For the dependent variable, among those who currently use cannabis, five modes of cannabis use in the past 30 days were evaluated. These modes included smoking (e.g., in a joint, bong, pipe, or blunt), vaping (e.g., using an e-cigarette-like vaporizer or other vaporizing devices), eating or drinking (e.g., in brownies, cakes, cookies, candy, or beverages like tea, cola, or alcohol), dabbing (e.g., using a dabbing rig, knife, or dab pen), and other methods. Each mode was coded as “yes” (1) if the participant used it, or “no” (0) if not. A binary coded multi-modal cannabis use was defined as participants reporting the use of two or more modes of cannabis (coded as 1), whereas single-modal use referred to participants reporting the use of only one mode (coded as 0). Furthermore, patterns of multi-modal of cannabis use were categorized based on the number of combinations of modes reported: single-mode (one mode), dual-mode (two combinations), triple-mode (three combinations), quadruple-mode (four combinations), and quintuple-mode (all five combinations).

Independent variables include sociodemographic factors including self-reported sex, race/ethnicity (categorized as non-Hispanic Black, Hispanic, non-Hispanic White, and other race/multiracial, which includes Asian, Native Hawaiian or other Pacific Islander, American Indian or Alaska Native, more than one race, and another race not listed, Hispanic, and do not know), age (18 to 24, 25 to 29, 30 to 34), marital status (married, not married), educational attainment (high school or less, some college or technical school, college or more), and sexual orientation (heterosexual, gay/lesbian/bisexual, something else/do not know).

To measure days of poor mental and physical health, respondents were asked two questions, respectively: (1) “Now thinking about your mental health, which includes stress, depression, and problems with emotions, for how many days during the past 30 days was your mental health not good?” and (2) “Now thinking about your physical health, which includes physical illness and injury, for how many days during the past 30 days was your physical health not good?” Based on their responses, the number of poor health days was categorized into three groups: 0 day, 1–13 days, and 14+ days, following the approaches used in prior studies [32,33].

Substance use measures were assessed using the established methods in prior studies [34]. Current cigarette smoking was defined having smoked at least 100 cigarettes in their lifetime and currently smoking “every day” or “some days”. The current use of smokeless tobacco and electronic cigarettes (e-cigarettes) was similarly defined based on participants reporting they used these products “every day” or “some days”. Cannabis use frequency in the past month was classified into four groups: 1–3 days, 4–10 days, 11–19 days, and 20–30 days, based on prior studies [35,36]. Alcohol consumption in the past 30 days was assessed by asking participants how many days they consumed alcohol in the past month (1–30 days). Binge drinking was defined as consuming five or more drinks on a single occasion for men and four or more drinks on a single occasion for women [37].

Data on the adoption of MCL and RCL for 23 states were obtained from publicly available sources (https://www.britannica.com/procon/recreational-marijuana-legalization-debate/Con-Quotes#ref393732 (assessed on 2 March 2025)). RCL and MCL were coded as binary variables (1: adopted, 0: not adopted) based on their adoption year. Participant state residence identifiers, represented by State Federal Information Processing Standards (FIPS) codes, were used to link participants to cannabis-related state-level legalization. By the end of 2023, MCLs had been adopted in 18 states, and RCLs in 9 states among the 23 states. The variation in cannabis legalization across these states enables the analysis of the effects of MCL and RCL on multi-modal cannabis use. This analysis focuses on the immediate effects of MCL and RCL adoption, rather than their lagged effects, due to the availability of two waves of BRFSS data in this study. Between 2022 and 2023, one state adopted MCL, and another adopted RCL. A simple difference-in-difference analysis was conducted separately to examine the effect of newly adopting MCL or RCL in these two states on multi-modal use during this period. The results indicated that changes in RCL adoption (*p* = 0.1394) and MCL adoption (*p* = 0.4876) did not significantly impact multi-modal use between 2022 and 2023.

### 2.3. Data Analysis

Univariate analyses were conducted to estimate the weighted prevalence of multi-modal cannabis use among young adults who reported current cannabis use in 23 states. These analyses also assessed the distribution of cannabis use across various patterns, including single-modal and multi-modal of use, categorized as dual (two modes), triple (three modes), quadruple (four modes), and quintuple (five modes). For participants reporting multi-modal cannabis use, specific combinations such as smoking combined with vaping or smoking combined with eating or drinking (edibles) were also analyzed. In addition to these prevalence estimates, summary statistics were calculated to describe the characteristics of the sample.

Multivariable logistic regression models were then used to examine associations between sociodemographic factors, mental and physical health, other substance use behaviors (e.g., cannabis use frequency, tobacco use, alcohol use), and state-level cannabis legalization policies (MCL and RCL) with the likelihood of engaging in multi-modal cannabis use, compared to single-modal use. This analysis utilized data from 23 states, each of which administered either the core or optional cannabis use modules in the 2022 and 2023 BRFSS surveys, but not both. Weighting adjustments followed BRFSS guidelines [38]. Specifically, for states administering core modules, the weight variable for core surveys was applied. For states administering optional modules, the corresponding optional module weight variables (e.g., LCPWTV2, LCPWTV3) were used. Since no state included both core and optional modules or multiple versions of optional modules, weights were applied without further adjustment. All analyses accounted for the complex survey design of BRFSS by incorporating stratification and clustering variables to ensure accurate variance estimation and population-level representativeness. Year variables are treated as fixed effects in the regression models. Statistical analyses were performed using SAS 9.4 software (SAS Institute, Cary, NC, USA), utilizing survey-specific procedures to adjust for weights, stratification, and clustering. Statistically significance was determined at *p* < 0.05.

## 3. Results

As shown in Table 1, among the U.S. young adults aged 18–34 years who currently use cannabis in 23 states (n = 7635; weighted n = 7,482,134), 56.8% reported multi-modal cannabis use, while 43.2% reported single-modal cannabis use. Among multi-modal users, dual-mode use was the most prevalent (54.8%), followed by triple-mode use (29.0%), quadruple-mode use (13.3%), and quintuple-mode use (2.9%).

Prevalence of each mode overall, single-modal use by each mode, and multi-modal use by combinations of modes among young adults who currently use cannabis is presented in Table 2. Among young adults who currently use cannabis (n = 7635), smoking is the most prevalent mode (83.2%), followed by comparable rates of vaping (41.9%) and edibles (40.9%), and dabbing (21.7%), and other forms (6.1%). Among young adults currently using cannabis who engaged in single-modal use (n = 3392), smoking is the most common (70.3%), followed by edibles (22.9%), vaping (5.9%), dabbing (0.8%), and other forms (0.4%) in this study sample. Among young adults who currently using cannabis who engaged in multi-modal use (n = 4243), dual-mode use was dominated by combinations such as smoking + vaping (22.0%) and smoking + edibles (17.3%). Other dual-mode combinations, such as vaping + dabbing or vaping + other, were reported at much lower rates. Among triple-mode users, the most common combinations included smoking + vaping + edibles (13.3%) and smoking + vaping + dabbing (10.8%). Quadruple-mode use, reported by 11.7% of multi-modal users, was predominantly a combination of smoking, vaping, edibles, and dabbing.

Table 3 presents summary statistics on multi-modal cannabis use among young adults who currently use cannabis, along with the results from the multivariable logistic regression analysis. Among U.S. young adults aged 18–34 years who engage in multi-modal cannabis use, 56.6% were male, 61.0% were non-Hispanic White, 71.4% were aged 18–29 years, and 80.5% had less than a college degree. Almost half of those engaging in multi-modal cannabis use also co-used e-cigarettes and alcohol with cannabis and reported frequent cannabis use, highlighting the strong overlap among multiple substances.

The multivariable logistic regression model of multi-modal cannabis use revealed several significant associations. Non-Hispanic African American young adults who currently use cannabis were less likely to report multi-modal use compared to their non-Hispanic White counterparts (AOR = 0.60, 95% CI = 0.42–0.86). Sexual minorities were more likely to engage in multi-modal cannabis use. Specifically, gay, lesbian, or bisexual individuals had higher odds of multi-modal cannabis use (AOR = 1.63, 95% CI = 1.26–2.12), and those who identified as other or were unsure of their sexual orientation also had an increased odds of engaging in multi-modal cannabis use (AOR = 1.97, 95% CI = 1.13–3.41). While self-perception on the days of impaired mental health was not significantly associated with multi-modal cannabis use, physical health was. Individuals who reported one to thirteen days (AOR = 1.30, 95% CI = 1.02–1.65) of impaired physical health had higher odds in engaging in multi-modal cannabis use compared to those who reported zero days of impaired physical health.

Co-use of substances with cannabis was a major factor in multi-modal cannabis use. Those who used cannabis for 4–10 days (AOR = 2.78, 95% CI = 1.98–3.89), 11–19 days (AOR = 4.56, 95% CI = 3.23–6.45), and 20–30 days (AOR = 6.46, 95% CI = 4.77–8.75) in the past month were more likely than those who used cannabis for 1–3 days to engage in multi-modal cannabis use in a frequency-dependent manner. Co-use of e-cigarettes (AOR = 1.86, 95% CI = 1.44–2.39) and alcohol (AOR = 1.43, 95% CI = 1.04–1.98) with cannabis and binge drinking (AOR = 1.28, 95% CI = 1.00–1.64) were also significantly associated with higher odds of multi-modal cannabis use, whereas current cigarette smoking and smokeless tobacco use were not significantly associated with multi-modal cannabis use. Furthermore, individuals residing in states with RCLs had significantly higher odds of engaging in multi-modal cannabis use compared to those in states without RCLs (AOR = 1.32, 95% CI = 1.03–1.70). No significant association was observed for MCL adoption.

## 4. Discussion

This secondary analysis of data from U.S. young adults in 23 states, derived from the 2022 and 2023 BRFSS, found that a substantial proportion (57%) of young adults who currently use cannabis and engaged in multi-modal cannabis use. Consistent with prior studies [9,10], smoking is the most common method to consume cannabis among young adults who use cannabis, with a significant preference for vaping or edibles. In our study sample, young adults who reported current cannabis use with single-modal use primarily smoked cannabis, followed by edibles and vaping. These findings differ slightly from prior epidemiological data, which shows higher rates of vaping compared to edibles [9,10]. However, the results of this study may reflect more recent trends, including the growing popularity and increasing sales of cannabis edibles [39], particularly among young adults, as shown in studies reporting higher rates of edibles use compared to vaping products based on more recent data [40,41]. Among young adults reporting current cannabis use with multi-modal use, the majority (84%) engaged in dual- or triple-mode combinations, with smoking, vaping, and edibles identified as the most common modes of use. These findings reflect a shifting landscape of cannabis use among U.S. young adults, where a significant proportion are diversifying their methods of administration rather than relying on single-modal use, potentially heightening exposure to increased cannabis-related harms and varied health risks.

Multi-modal cannabis use among young adults varied by race/ethnicity and sexual orientation. Non-Hispanic African American individuals who reported current cannabis use had lower odds of engaging in multi-modal use compared to their non-Hispanic White counterparts. Conversely, individuals identifying as gay, lesbian, bisexual, something else, or uncertain about their sexual orientation had higher odds of multi-modal use than heterosexual individuals. These patterns can be attributed to cultural preferences and motivations for cannabis use. For instance, African American young adults primarily consume cannabis through smoking methods like blunts, while vaping and edibles are less common [42], which may reduce their likelihood of multi-modal use. In contrast, sexual minorities tend to engage with a variety of product types of cannabis and rely on cannabis as a coping mechanism for stress and mental health issues exacerbated by minority stress [18]. This heightened use increases their risk of cannabis use disorder (CUD) and contributes to broader disparities in substance use behaviors [18,43]. The findings of this study provide robust evidence of disparities in multi-modal cannabis use across certain subgroups using a nationally representative sample of U.S. young adults. These insights underscore the importance of considering sociodemographic factors and subgroup-specific risks in cannabis research and intervention efforts aimed at addressing substance use-related health inequities.

Physical health status emerged as a significant predictor of multi-modal cannabis use among young adults. This finding suggests that young adults with poor physical health may turn to multi-modal cannabis use as a self-management strategy to alleviate symptoms associated with chronic pain or other physical conditions [23,36,44]. Cannabis is often viewed as a natural or less harmful alternative to traditional pain management methods [36], which may motivate individuals to experiment with multiple modes of administration. However, it is important to note that these findings do not establish a causal relationship between negative physical health symptoms and multi-modal cannabis use, due to the limitations of the cross-sectional design of this study. Alternatively, multi-modal cannabis use may exacerbate physical health problems, as increased exposure to cannabis could have adverse health effects, particularly among individuals with pre-existing physical conditions [45].

Cannabis use intensity and co-use with other substances, such as e-cigarettes and alcohol, significantly increased the likelihood of multi-modal cannabis use among young adults. The more frequent cannabis use was strongly associated with higher odds of multi-modal use, suggesting frequency-dependent pattern in which increased cannabis use correlates with a greater likelihood of using multiple consumption methods [46]. Although co-use of cannabis with cigarettes and smokeless tobacco was not significantly associated with multi-modal use in this study, co-use with e-cigarette vaping was notably linked to a higher likelihood of engaging in multi-modal use. This finding aligns with recent evidence indicating that vaping has become the predominant method of nicotine consumption among young adults [47], which may facilitate co-administration behaviors, such as co-vaping of cannabis and e-cigarettes [48,49] and contribute to the expansion of dual- and poly-mode cannabis use. Furthermore, alcohol use and binge drinking also increased the risk of multi-modal use, underscoring the growing interplay among cannabis, tobacco, and alcohol among young adults. To effectively reduce multi-modal cannabis use among this vulnerable population, it is essential to address the co-use of other substances rather than focusing on cannabis use in isolation.

Consistent with prior research on cannabis use in states with cannabis legalization [26,27,28,29], our study found that RCL was significantly associated with increased odds of multi-modal cannabis use among young adults, while MCL showed the null effect. The limited impact of MCL may be attributed to the lower prevalence of medical cannabis use among young adults compared to older adults [50] and the stricter regulations, which require a prescription and a doctor’s recommendation and restrict access to a narrower range of products than the recreational market [51]. This suggests that although cannabis use is more common among young adults, the legal avenues for medical cannabis access may not significantly influence patterns of multi-modal use. In contrast, young adults residing in states with RCL were more likely to engage in multi-modal cannabis use in this study. RCL typically expands access to a broader range of high-potency products and diverse consumption methods, including vaping or dabbing concentrates [52], which may encourage young adults to experiment cannabis use with different and multiple modes of use. Furthermore, the increased availability and accessibility of cannabis through recreational retailers in RCL states are linked to higher rates of cannabis use and greater intensity of use, including cannabis use disorder [53]. This heightened cannabis use and disorder are often associated with multi-modal cannabis use [11,12], as individuals may be more inclined to explore various modes of consumption when products are readily available.

Although this study has several strengths, including the use of the representative sample from 23 states in the U.S. and a multi-modal cannabis use model with a comprehensive range of sociodemographic, health, substance use, and contextual factors such as MCL and RCL, it also has limitations. First, the use of two waves of cross-sectional data limits our ability to track changes in multi-modal cannabis use or the effects of changes in cannabis legalization on multi-modal cannabis use over time, preventing us from making causal inferences. Second, the self-reported nature of the data may introduce recall or social desirability bias [54], particularly for sensitive behaviors like cannabis use or co-use with other substances. Third, while the study includes data from 23 states, the findings may not be fully generalizable to all U.S. young adults, particularly those in states without cannabis legalization. Additionally, sexual orientation data were not collected in four states (Maine, Mississippi, Nebraska, and Oregon), potentially limiting the representativeness of this variable in our analyses. Fourth, this study was limited in its ability to directly assess the effects of variability in various provisions of MCLs and RCLs (e.g., product availability, dispensary regulations) [53,55] on multi-modal cannabis use across states. Additionally, the constraints of the secondary analysis using BRFSS data meant that measures of perceptions of cannabis harms and specific product availability such as high-potency products were not included, which could contribute to the association between cannabis legalization and modes of cannabis use [52,56]. Therefore, future studies are needed to examine how the nuanced variability in MCLs and RCLs, which reflect a complex regulatory landscape, as well as individual’s perceptions of drug harms and actual availability, dynamically contribute to the relationships between cannabis legalization and multi-modal cannabis use patterns among young adults. Lastly, the BRFSS measures of cannabis use modes have limitations in accurately identifying and differentiating between the specific devices used for cannabis consumption, especially when the similar device can be used for multiple modes. For example, we were unable to differentiate between vaping cannabis using a vaporizer and dabbing by inhaling vaporized cannabis concentrates, as both methods involve the inhalation of vaporized cannabis products.

The findings of this study have implications for cannabis research and practice. Continuous surveillance is crucial to monitor evolving patterns of multi-modal cannabis use, particularly in relation to emerging cannabis products and their intersection with physical and mental health outcomes, as well as co-use with other substances. Although this study does not directly compare the risks of multi-modal versus single-modal cannabis use, ongoing research is necessary to elucidate these comparative risks. This continuous monitoring will be essential for generating robust evidence to inform policy and intervention strategies aimed at mitigating potential harms of multi-modal cannabis use among young adults. Public health campaigns should raise awareness of the risks associated with using multiple consumption methods, particularly in states with RCLs. Policymakers may consider regulating the availability of high-potency products and restricting marketing that promotes multi-modal use. Furthermore, tailored interventions should target high-risk groups, such as sexual minorities and individuals with poor physical health, and integrate cannabis education into broader substance use prevention efforts that address co-use with substances like alcohol and nicotine vaping. This approach will help reduce disparities related to cannabis use and associated substances.

## 5. Conclusions

In conclusion, this study provides evidence of the substantial prevalence of multi-modal cannabis use and diversification of consumption methods among U.S. young adults who currently use cannabis, with dual- and triple-mode use being the most common patterns. Key factors associated with higher odds of multi-modal use include sexual minority status, poor physical health, frequent cannabis use, and co-use with substances such as e-cigarettes and alcohol. Additionally, RCL was significantly associated with higher odds of multi-modal cannabis use, highlighting the critical role of policy environments in shaping cannabis use behaviors. These findings underscore the need for targeted prevention strategies aimed at young adults at elevated risk of multi-modal cannabis use, addressing both individual risk factors and broader policy influences to mitigate potential cannabis-related harms.

## Figures and Tables

**Table 1:** Prevalence of single-modal use, multi-modal use, and patterns of multi-modal cannabis use among the U.S. young adults (18–34 years) who currently use cannabis in 23 states: 2022–2023 BRFSS (n = 7635; weighted n = 7,482,134).

	Unweighted n	Weighted%	95% CI
Single-modal use	3392	43.2	41.2–45.1
Multi-modal use ^a^	4243	56.8	54.9–45.1
Dual-mode	2305	54.8	52.3–57.4
Triple-mode	1266	29.0	26.7–31.3
Quadruple-mode	574	13.3	11.5–15.0
Quintuple-mode	98	2.9	2.0–3.8

^a^ The weighted %s of dual-, triple-, quadruple-, and quintuple-mode cannabis use were calculated using young adults who reported cannabis use and engaged in multi-modal use (unweighted n = 4243) as the denominator (unweighted n = 4243). CI = Confidence interval.

**Table 2:** Prevalence of each mode among U.S. young adults (18–34 years) who currently use cannabis (n = 7635), those who engaged in single-modal use (n = 3392), and those who engaged in multi-modal use (n = 4243) by 25 specific combinations of cannabis use mode patterns in 23 states: 2022–2023 BRFSS (n = 7635; weighted n = 7,482,134).

Overall	Unweighted n	Weighted%	95% CI
Smoking	6203	83.2	81.9–84.6
Vaping	2988	41.9	40.0–43.8
Edibles	3265	40.9	39.0–42.8
Dabbing	1698	21.7	20.1–23.3
Other	432	6.1	5.2–7.1
Single-modal cannabis use			
Smoking	2300	70.3	67.8–72.8
Vaping	216	5.9	4.8–7.0
Edibles	835	22.9	20.5–25.2
Dabbing	19	0.8	0.2–1.4
Other	22	0.4	0.1–0.6
Multi-modal cannabis use			
Dual-mode			
Smoking + Vaping	786	22.0	19.7–24.3
Smoking + Edibles	810	17.3	15.5–19.1
Smoking + Dabbing	301	5.9	4.8–7.1
Smoking + Other	103	3.2	2.1–4.3
Vaping + Edibles	189	3.9	2.9–4.9
Vaping + Dabbing	56	1.3	0.7–2.0
Vaping + Other	13	0.3	0.0–0.5
Edibles + Dabbing	12	0.1	0.0–0.2
Edibles + Other	34	0.6	0.3–1.0
Dabbing + Other	1	0.0	0.0–0.1
Tripple-mode			
Smoking + Vaping + Edibles	537	13.3	11.6–15.0
Smoking + Vaping + Dabbing	472	10.8	9.2–12.4
Smoking + Vaping + Other	27	0.6	0.3–1.0
Smoking + Edibles + Dabbing	153	2.8	2.1–3.5
Smoking + Edibles + Other	31	0.5	0.3–0.7
Smoking + Dabbing + Other	14	0.4	0.1–0.7
Vaping + Edibles + Dabbing	24	0.3	0.2–0.5
Vaping + Edibles + Other	4	0.2	0.0–0.5
Vaping + Dabbing + Other	3	0.0	0.0–0.1
Edibles + Dabbing + Other	12	0.1	0.0–0.2
Quadruple-mode			
Smoking + Vaping + Edibles + Dabbing	493	11.7	10.1–13.4
Smoking + Vaping + Edibles + Other	30	0.6	0.3–1.0
Smoking + Vaping + Dabbing + Other	37	0.8	0.4–1.3
Vaping + Edibles + Dabbing + Other	3	0.0	0.0–0.1
Quintuple-mode			
Smoking + Vaping + Edibles + Dabbing + Other	98	2.9	2.0–3.8

CI = Confidence interval.

**Table 3:** Summary statistics on multi-modal cannabis use among the U.S. young adults (18–34 years) who currently use cannabis and multivariable regression of multi-modal use with sociodemographic factors, mental and physical health, substance use and cannabis legalization policies: 2022–2023 BRFSS (n = 7635; weighted n = 7,482,134).

Variables	Unweighted n (n = 4243) ^a^	Weighted%	AOR (95% CI)
Sex			
Male	2541	56.6	1.19 (0.94–1.50)
Female	1702	43.4	Ref
Race/Ethnicity			
Non-Hispanic Black	280	12.2	0.60 (0.42–0.86) **
Non-Hispanic Others	478	11.8	1.38 (1.95–2.00)
Hispanic	527	13.3	1.07 (0.78–1.46)
Do not know	77	1.6	0.88 (0.37–12.10)
Non-Hispanic White	2862	61.0	Ref
Age			
Age 18 to 24	1701	47.0	1.27 (0.94–1.72)
Age 25 to 29	1256	24.4	1.16 (0.88–1.54)
Age 30 to 34	1286	28.5	Ref
Family income			
<USD 25,000	536	14.4	0.98 (0.70–1.37)
≥$25,000	2992	85.6	Ref
Educational Attainment			
Highschool or less	1784	48.6	Ref
Some college or technical school	1271	31.9	0.84 (0.64–1.11)
College graduate or more	1184	19.5	1.08 (0.81–1.46)
Marital status			
Married	1461	32.4	0.94 (0.74–1.20)
Not married	2753	67.7	Ref
Sexual Orientation			
Gay, lesbian, or bisexual	790	26.4	1.63 (1.26–2.12) ***
Something else or do not know	190	7.6	1.97 (1.13–3.41) *
Heterosexual	1976	65.9	Ref
Days of impaired mental health			
0 day	865	21.3	Ref
One to thirteen days	1723	40.7	0.98 (0.71–1.34)
Fourteen+ days	1579	38.0	0.97 (0.69–1.36)
Days of impaired physical health			
0 day	2057	48.8	Ref
One to thirteen days	1636	39.7	1.30 (1.02–1.65) *
Fourteen+ days	480	11.5	1.49 (0.97–2.27)
Cannabis use frequency			
1–3 days	1812	22.3	Ref
4–10 days	1361	17.0	2.78 (1.98–3.89) ***
11–19 days	1128	14.6	4.56 (3.23–6.45) ***
20–30 days	3334	46.1	6.46 (4.77–8.75) ***
Current cigarette smoking	889	20.0	0.97 (0.71–1.31)
Current smokeless tobacco use	252	5.6	1.46 (0.92–2.33)
Current e-cigarette use	1600	41.5	1.86 (1.44–2.39) ***
Past 30-day alcohol use	3245	77.8	1.43 (1.04–1.98) *
Binge drinking	1873	43.8	1.28 (1.00–1.64) *
MCL adoption	3371	87.1	0.81 (0.61–1.08)
RCL adoption	2424	53.5	1.32 (1.03–1.70) *
Year			
Year of 2022	2257	56.6	Ref
Year of 2023	1986	43.4	1.06 (0.85–1.32)

^a^ Unweighted sample size for those who reported multi-modal cannabis use among the U.S. young adults aged 18–34 years who currently use cannabis (unweighted n = 7635). The reference group comprises participants who reported “no” responses for current cigarette smoking, current smokeless tobacco use, current e-cigarette use, past 30-day alcohol use, binge drinking, and the adoption of MCL and RCL variables. E-cigarette = electronic cigarette. MCL = Medical cannabis law. RCL = Recreational cannabis law. AOR = Adjusted odds ratio. CI = Confidence interval. Ref = Reference. * *p* < 0.05, ** *p* < 0.01, *** *p* < 0.001.

## Data Availability

Deidentified Behavioral Risk Factor Surveillance System (BRFSS) data methodology and the BRFSS questionnaire are publicly available form the Center for Disease Control and Prevention (CDC) (https://www.cdc.gov/brfss/index.html (assessed on 2 March 2025)).
